# Multilevel Fine Fault Diagnosis Method for Motors Based on Feature Extraction of Fractional Fourier Transform

**DOI:** 10.3390/s22041310

**Published:** 2022-02-09

**Authors:** Hao Wu, Xue Ma, Chenglin Wen

**Affiliations:** 1 School of Automation, Hangzhou Dianzi University, Hangzhou 310018, China; haowu@hdu.edu.cn (H.W.); xuema@hdu.edu.cn (X.M.); 2 School of Automation, Guangdong University of Petrochemical Technology, Maoming 525000, China

**Keywords:** fault diagnosis, motor, fractional Fourier transform, feature extraction

## Abstract

Motors are the main driving power for equipment operation, and they are also a major factor to promote the development of the motor and the load it drives and its motor control system toward a low-carbon future, reduce carbon emissions, and improve the industrial economy and social economic efficiency. Due to high-speed, long-period, and heavy-load operation, various faults occur; since the existing integer-order Fourier transform methods have not enough able to detect fractional-order faults and lack robustness, it is difficult to realize the fine diagnosis of motor faults, which reduces the safety and reliability of the motor control system. For this reason, on the basis of the powerful extraction ability of the fractional Fourier transform (FRFT) for micro fault features, especially the extraction ability to fit fractional frequency domain faults, this paper intends to establish a multilevel fine fault diagnosis method for fractional-order or integer-order faults. Firstly, this is accomplished by performing the fractional Fourier transform on the acquired data with faults and feature extraction in the multilevel fractional frequency domain and then optimizing the feature extraction model. Secondly, one further step search method is established to determine the projection direction with the largest fault feature. Thirdly, taking the extracted multilevel fault features as input, a multilevel fine fault diagnosis method based on the SVM model is established. Finally, three typical digital simulation examples and actual operating data collected by the ZHS-2 multifunctional motor test bench with a flexible rotor are employed to verify the effectiveness, robustness, and accuracy of this new method. The main contribution and innovation of this paper are that the fractional Fourier transform method based on time domain and frequency domains is introduced. This method can extract the small fault features in the maximum projection direction of the signal in the fractional domain, but detection with other time–frequency methods is difficult; the extracted multilevel fault features are used as input, and the corresponding fault diagnosis model is established, which can improve the accuracy of fault detection and ensure the safe and reliable operation of industrial equipment.

## 1. Introduction

As people’s demand for economy continues to grow, so does the demand for industrial production. Electrical equipment will inevitably need to work for a long time during the industrial production process. However, the possibility of equipment failure under long-term high-load continuous operation is greatly increased. Because the motor is the main driving force for the operation of the motor control system, once the electrical equipment fails, the motor control system will have low motor conversion efficiency and paralysis of the entire power system. In severe cases, it will even cause huge economic losses and casualties. Therefore, real-time fault monitoring of electrical equipment during industrial production is very necessary [1,2,3,4,5,6]. Fault diagnosis is an important technology to ensure the safe and stable operation of industrial systems; when the diagnosis technology is more advanced, it can detect minor faults in an early and accurate manner. Minor faults usually refer to the fault characteristics that are not obvious and are easily hidden by noise signals in the working environment or refer to a failure that has a very small impact on the safe operation of the industrial equipment system at the initial stage of the failure, but during the operation of the equipment, it will cause a destructive effect on the safe operation of the industrial equipment system. In 2012, Li et al. reviewed the research status of minor fault diagnosis at home and abroad in the literature [7]; in the context of industrial big data, data-driven fault diagnosis methods have gradually become a hot research direction. In 2016, Wen et al. classified “data-driven fault diagnosis methods” into three categories: statistical analysis methods, signal processing methods, and artificial intelligence methods [8]. The fractional Fourier transform proposed in this paper is one of the important directions in signal processing methods [9,10,11], and it was first widely used in radar, communications, information security, and other fields [12,13,14]. In recent years, the FRFT has been researched in the detection and parameter estimation of chirp signals [15,16], and in [17], FRFT was used for early fault diagnosis of gearboxes.

The current analysis methods for motor faults are mainly based on feature extraction in the time domain and frequency domain. The earliest method is the analysis of the fault signal of a rolling bearing from the perspective of the time domain. It is complicated to compare various complex parameters of the signal and analyze the given standard signal and the detected signal to detect the fault characteristics. Since this method takes a lot of time to compare, the final result is not good [18]. Frequency domain analysis methods, such as the classic Fourier transform method, can more clearly reflect the characteristics of the fault signal in the frequency domain, but it is not enough to analyze the fault signal in the time domain or the frequency domain alone. The short-time Fourier transform solves the above problems to a certain extent [19,20]. It can analyze the detected signal from both the time domain and the frequency domain at the same time. However, the detection of the fractional fault signal by this method is not obvious. It is difficult to find the maximum projection direction for fractional fault characteristics, so the maximum fault characteristic value under the optimal projection direction cannot be obtained. It takes a lot of later work to detect the fractional fault. Wavelet transform is a new transform analysis method. It inherits and develops the idea of localization of short-time Fourier transform and at the same time overcomes the shortcomings of window size that does not change with frequency and can provide a “time–frequency” window that changes with frequency; it is an ideal tool for signal time–frequency analysis and processing [21,22,23]. The Hilbert–Huang transform method mainly consists of two parts: empirical mode decomposition and Hilbert spectral analysis. The empirical mode decomposition method is an adaptive and efficient data decomposition method, and since the decomposition is based on a local time scale, it is suitable for nonlinear and nonstationary processes [24,25,26,27]. Important research on wavelet and Hilbert–Huang analysis for failure detection has been conducted since 2008 by European and American researchers; by analyzing and improving the principles of wavelet and Hilbert–Huang, they applied them to the field of fault diagnosis, provided new diagnostic methods, and promoted the development of the field of fault diagnosis [28,29,30,31,32]. When processing chirp signals, not only time-domain waveforms but also spectrum analysis are used in signal processing, and related researchers have proposed different solutions [33,34,35]. The fractional Fourier transform can not only analyze the signal in the time domain and frequency domain, but also obtain the direction of the maximum projection of the signal in the fractional domain by rotating the signal; a multilevel fault diagnosis method is constructed with different transformation orders, but due to the precision selection of the transformation order and the need for a lot of calculations when transforming, there are still some problems in the application of this method in real-time fault detection. In addition, the existing methods cannot achieve a good effect when processing signals containing both integer-order faults and fractional-order faults [36,37,38]. In multilevel fault diagnosis methods, new methods using adaptive processing of signal samples have been proposed one after another, which can detect and locate multiple faults more efficiently and reliably than traditional fault diagnosis methods [39,40]. As the structure of the neural network gets deeper and deeper, the gradient descent algorithm can use the Kalman filter to adaptively update the neural network. Big data causes a serious problem of rich data and lack of knowledge, that is, although the number of samples is large, there are few labeled samples endowed with knowledge. This phenomenon seriously hinders the promotion of deep convolutional neural network models in real life [41,42,43,44,45].

This paper uses the FRFT algorithm to analyze the chirp signal from a fault signal in the time–frequency domain. The chirp signal is a frequently occurring fractional fault signal. The prerequisite for detecting a fractional fault is the chirp signal also being able to be detected. FRFT has a strong ability to detect chirp signals under strong noise and other interference. The fault feature values in the detection signal are extracted and input into the SVM model through FRFT, and a more accurate diagnostic model for fractional fault detection in bearings can be obtained. The simulation experiment part of this paper further demonstrates the effectiveness of this method for fractional fault detection, improves the safety and reliability of the motor control system, saves energy and reduces consumption, and achieves low carbon. The content of this article is arranged as follows: Section 2 introduces the chirp signal. Section 3 introduces the background and definition of FRFT and then introduces the principle and method of FRFT extraction of target components and the selection of the optimal transformation order of FRFT. Section 4 introduces the fault diagnosis method based on SVM. Section 5 presents the experimental simulation results and analysis. Section 6 is the summary and prospect.

## 2. Chirp Signal

A chirp signal can also be called a linear frequency modulation (LFM) signal, which is a classic nonstationary signal. It is a professional term for coded pulse technology in communication technology and is widely used in communications, radar positioning, sonar, and other fields [46]. For example, in radar positioning technology, it can increase the width of radiofrequency pulses, increase the average emission power, and increase the distance between communications while maintaining sufficient signal spectrum width to maintain the range resolution of the radar. When many industrial devices such as motor rotors are in operation, the characteristic frequency of the fractional-order fault changes very slowly. It tends to change linearly, which is very similar to the chirp signal. The basic mathematical expression of the chirp signal is as follows:

(1) $$x(t)=Aexp(j2\pi (f_{0}t+1/2f_{m}t^{2})),0\leq t\leq T$$

where A is the amplitude of the chirp signal; j is the complex symbol; $f_{0}$ is the starting frequency and $f_{m}$ is the FM frequency, both of which are constants, with the unit of Hz/s; *T* is the time width of the pulse; and x(t) is the final expression of the chirp signal. By derivation of the phase term in Equation (1), the instantaneous frequency of the chirp signal and the relationship of its frequency with time can be obtained, as shown in Equation (2), where θ(t) is the twist angle function as the signal propagates over time.

(2) $$f(t)=\frac{1}{2\pi }\frac{d\theta (t)}{dt}=f_{0}+f_{m}t$$

**Figure 1:**
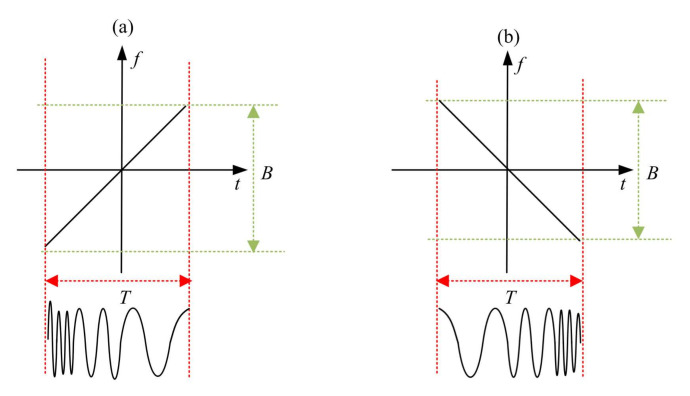
Time–frequency relationship diagram and waveform diagram of chirp signal: (**a**) positive chirp signal; (**b**) negative chirp signal.

Figure 1 shows the time–frequency relationship diagram and waveform intent of the chirp signal. As shown in Figure 1a, when the FM frequency $u_{0}$ of the chirp signal is positive, the signal is called a positive chirp signal. Within one cycle, the bandwidth of the signal is B, and its instantaneous frequency increases from $f_{0}$ to $f_{0}+f_{m}t$; that is, the frequency increases linearly with the increase in time, and the signal waveform appears from sparse to dense in the time domain. As shown in Figure 1b, when the FM frequency $u_{0}$ of the chirp signal is negative, the signal is called a negative chirp signal. Within one cycle, the bandwidth of the signal is B, and its instantaneous frequency is determined by $f_{0}$ decreasing to $f_{0}+f_{m}t$; that is, the frequency decreases linearly with the increase in time, and the signal waveform appears from dense to sparse in the time domain.

## 3. The Proposed Method

### 3.1. Background and Definition of FRFT

Fractional Fourier transform (FRFT) was not taken seriously from 1929 until 1980, when V. Namias studied the Fourier transform from the perspective of eigenvalues and eigenfunctions and defined the fractional power form of the traditional Fourier transform as the fractional Fourier transform. The FRFT was first used to solve the Schrodinger equation in quantum mechanics, and then it was introduced into the field of optics by Mendlovic and others, and it was the first to be applied. Now the FRFT is used in Fourier optics, signal analysis, and research on radar tracking-related fields and has attracted the attention of scholars from various countries [47].

FRFT is a method of transforming a signal from the time domain to the fractional domain. The mathematical expression of the positive and negative transformation is:

(3) $$X_{p}(u)=\left\{\left.F_{p}[x(t)]\right\}\right.(u)=\int_{-\infty }^{+\infty }K_{p}(t,u)x(t)dt$$

(4) $$x(t)=\int_{-\infty }^{+\infty }K_{-p}(t,u)X_{p}(u)du$$

Equation (3) is a positive transformation, and Equation (4) is an inverse transformation. $F_{p}$ is the FRFT operator; x(t) is the input signal; u is the fractional domain; $X_{p}(u)$ is the value of the signal after FRFT; and $K_{p}(t,u)$ is the kernel function of the FRFT, which can be expressed as:

(5) $$K_{p}(t,u)=\left\{\begin{array}{l} A_{p}exp(j\pi \frac{\left(u^{2}+t^{2}\right)}{2}\cot\frac{p\pi }{2}-j\pi \frac{2ut}{\sin p\pi })\;p\neq 2n\\ \delta (t-u)\;\;\;\;\;\;\;\;\;p=4n\\ \delta (t+u)\;\;\;\;\;\;\;\;\;p=4n\pm 2 \end{array}\right.$$

In the above formula, $A_{p}=\sqrt{1-j\cot\frac{p\pi }{2}}$ is the amplitude of the FRFT; α=pπ/2 is the rotation angle of the FRFT; p is the transform order of FRFT; when p=4n, $K_{p}(t,u)=\delta (t-u)$; when p=4n+2, $K_{p}(t,u)=\delta (t+u)$; n is an integer; δ(t+u) represents adding a phase; and δ(t−u) represents subtracting a phase.

After selecting the specific parameters of the kernel function, the specific FRFT form is also determined by transforming the order p and the rotation angle α. When p=0, according to the formula α=0, Equation (3) becomes $X_{p}(u)=x(t)$, that is, not rotated, and it is still the signal itself; when p=1, $\alpha =\pi /2,\;A_{\alpha }=1$, and then Equation (3) becomes $X_{p}(u)=\int_{-\infty }^{+\infty }exp(-j2\pi ut)x(t)dt$; according to $X_{p}(u)$, it can be seen that this is the classical Fourier transform (FT), so FRFT can be understood as a generalized Fourier transform; when p=4, α=2π, and then Equation (3) becomes $X_{p}(u)=x(t)$ again. In order to simplify the calculation of the formula and unify the expression of variables, after variable substitution $u=u/\sqrt{2\pi }$ and $t=t/\sqrt{2\pi }$, Equation (3) can be further expressed as:

(6) $$X_{p}(u)=\left\{\begin{array}{l} \sqrt{\frac{1-j\cot\alpha }{2\pi }}\int_{-\infty }^{+\infty }exp(j\frac{\left(u^{2}+t^{2}\right)}{2}\cot\alpha -j\frac{ut}{\sin\alpha })x(t)\;\alpha \neq n\pi \\ x(t)\;\;\;\;\;\;\;\;\;\;\;\;\;\;\;\alpha =2n\pi \\ x(-t)\;\;\;\;\;\;\;\;\;\;\;\;\;\;\alpha =(2n\pm 1)\pi \end{array}\right.$$

In the above formula, x(t) is the signal equal to the original signal after transformation, x(−t) is the value of the signal after transformation, and the transformed signal and the original signal are symmetrical at the origin. By deriving Equation (3), it can be seen that the FRFT is defined by the transformation order p or the rotation angle α as a parameter, so the definition with p (or α) as the parameter takes 4 or 2π as the period. Therefore, we only need to examine the interval $p\in \left[-2,2\right]$ (or α ∈ (−π, π)). The definition of this segmentation artificially makes the kernel function $K_{p}(t,u)$ take the value of all continuous p.

### 3.2. The Method and Principle of Extracting Target Components by FRFT

When an industrial device such as a motor rotor has a fractional-order fault during operation, due to the fractional-order fault being different from the integer-order fault, the commonly used signal processing methods cannot find the maximum projection direction and fault characteristic value in this optimal projection direction when extracting the characteristics of the fractional fault. Therefore, under strong background noise, determining how to extract the maximum fault characteristic value in a fractional-order fault will be a key scientific problem studied in this paper. According to this technical problem, the industrial fault signal data are analyzed by FRFT, and the characteristic value of the fractional fault under the background of noise is extracted to achieve the effect of fractional fault analysis.

**Figure 2:**
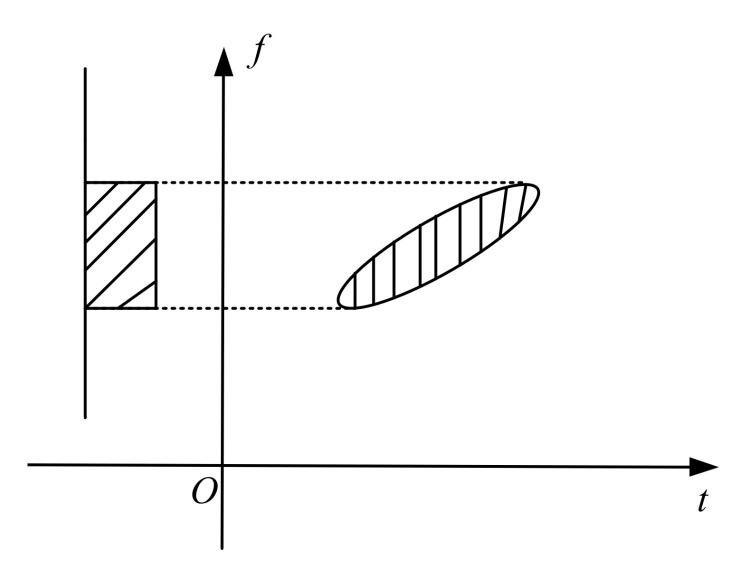
Projection of chirp signal on Fourier domain [48].

**Figure 3:**
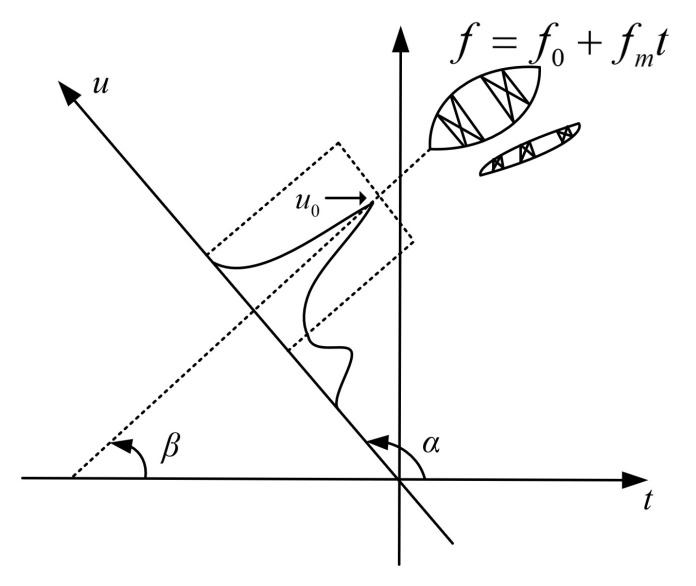
FRFT extraction of LFM components [48].

The chirp signal is obliquely elliptical in the time–frequency distribution. Since the classical Fourier transform directly projects the time–frequency distribution of the chirp signal on the frequency axis, this results in a wider projection signal spectrum of the LFM signal on the frequency axis, and the signal energy cannot be gathered very well and cannot detect the signal well, as shown in Figure 2 [48]. The time–frequency distribution diagram of the two-component chirp signal is shown in Figure 3 [48]. The angle between the time–frequency distribution of one component LFM signal and the time axis is β. Point $u_{0}$ in Figure 3 represents the peak at an energy gathering point obtained after FRFT under the maximum projection direction of the signal in the fractional domain; it is the extracted tiny fault feature value of the signal in the fractional domain. At this point, according to the definition of the FRFT, the FRFT is equivalent to rotating the signal around the origin on the time–frequency plane and then representing it in the fractional domain formed after the rotation. The projection curve of the LFM signal can be obtained from the mathematical expression of FRFT. FRFT can also be understood as a representation method in the fractional domain formed by rotating the coordinate axis counterclockwise around the origin by any angle in the time–frequency plane. As long as the rotation angles α and β of FRFT are orthogonal, the LFM component can be gathered at the $u_{0}$ point of Figure 3 in the fractional Fourier domain, which is the maximum value obtained after FRFT of the signal and the target components to be extracted. The fractional domain aggregation point $u_{0}$ is taken as the center for filtering processing to obtain a fractional Fourier domain distribution with a high concentration of energy of the LFM component so that the chirp signal can be separated in the presence of multiple components, and finally, through an operation such as inverse transformation, the signal is extracted [48].

The classical Fourier transform transforms the viewing angle from the time domain to the frequency domain. The FRFT rotates the signal at any angle from the angle of viewing the time–frequency plane and then analyzes the information from the perspective of observing the fractional domain. It can be seen from the analysis that the reason for the FRFT of information is that most of the information is a nonstationary signal, and the FT alone is not enough to analyze its salient features. The use of fractional Fourier transform is mainly to select the most concentrated angle for analysis. That is, the result with the largest amplitude is selected from the results obtained by different fractional orders, and then the fractional order in which this result exists is the optimal order. It can be seen from Figure 3 that the relationship between the transformation order p, the rotation angle α, and the frequency modulation frequency $f_{m}$ is:

(7) $$\left\{\begin{array}{l} \beta =\arctan(f_{m})\\ \alpha =\pi /2+\beta =\pi /2+\arctan(f_{m})\\ p=2\alpha /\pi =1+2\arctan(f_{m})/\pi \end{array}\right.$$

The fast algorithm is the basis for the successful application of the FRFT in signal processing. This paper adopts an efficient and accurate calculation of the FRFT algorithm proposed by Ozaktas [10]; the calculation process of the specific FRFT is decomposed as follows: For convenience, here is the redefinition of the FRFT. The redefined fractional Fourier transform formula is adopted here so that the calculation amount of each variable can be simplified in the following calculation process. The specific formula is as follows:

(8) $$X_{p}(u)=A_{\alpha }\int_{-\infty }^{+\infty }exp(j\pi (u^{2}\cot\alpha -2ut\csc\alpha +t^{2}\cot\alpha ))x(t)dt$$

Here, $A_{\alpha }=\exp(-j\pi \mathrm{sgn}(\sin\alpha )/4+j\alpha /2)/{\left|\sin\alpha \right|}^{1/2},\alpha =p\pi /2$. Assuming the order $p\in \left[-1,1\right]$, the above formula is divided into the following four operations:

Step 1: Select a chirp signal $\exp(-j\pi t^{2}\tan(\alpha /2))$ to modulate the signal x(t) to obtain the modulated signal g(t).

(9) $$g(t)=\exp(-j\pi t^{2}\tan(\alpha /2))x(t)$$

Step 2: Choose another chirp signal $\exp(-j\pi t^{2}\csc\alpha )$ to convolve with the signal g(t) to obtain the signal $g^{\prime }(u)$. The reason why another LFM signal is used to convolve the signal is that the convolution of two signals of the same type can decompose the signal into the sum of the impulse signals, and the impulse response of the system can be used to solve the zero-state response of the system to a signal. At the same time, this method is used to solve the problem of the existing fractional-order methods not being able to detect integer-order faults well, and it is an indispensable step.

(10) $$g^{\prime }(u)=\int_{-\infty }^{+\infty }\exp(-j\pi {\left(u-t\right)}^{2}\csc\alpha )g(t)dt$$

Step 3: Use the chirp signal $\exp(-j\pi u^{2}\tan(\alpha /2))$ to modulate the signal $g^{\prime }(u)$ again:

(11) $$x(u)=\exp(-j\pi u^{2}\tan(\alpha /2))g^{\prime }(u)$$

Step 4: Multiply the intermediate result obtained in step 3 by the complex coefficient $A_{\alpha }$ to obtain the final fractional Fourier transform value $X_{p}(u)$.

(12) $$X_{p}(u)=A_{\alpha }x(u)$$

**Figure 4:**
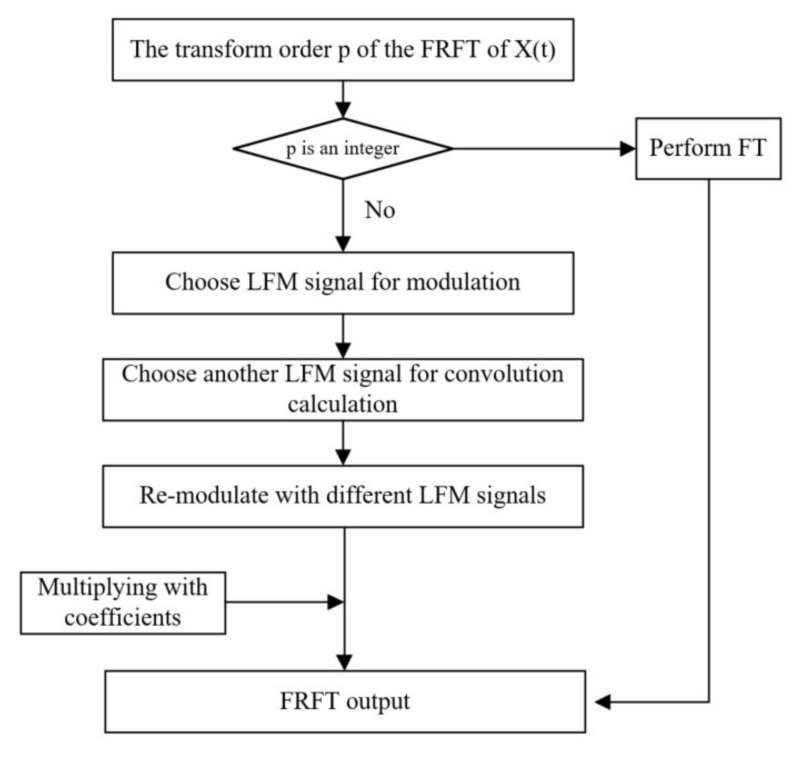
FRFT extraction of target components.

The algorithm decomposes the complex integral expression of the FRFT into several simple steps and then undergoes discretization processing to obtain a discrete convolution expression and perform calculations. In order to show the algorithm more intuitively, its flowchart is shown in Figure 4.

### 3.3. Selection of Optimal Transformation Order of FRFT

The order change interval of the FRFT is (0,2). When the transformation order takes different values in this interval, different transformation values will be obtained. There is a maximum value among these transformation values; the transformation order corresponding to the maximum value of the transformation is called the optimal transformation order. FRFT can extract the maximum eigenvalue of the signal under the optimal transformation order. When we use FRFT to extract the maximum fractional fault eigenvalue of the fault signal, training a diagnostic model with these eigenvalues can greatly improve the diagnostic accuracy of the model compared with the traditional diagnostic model, especially when detecting fractional-order faults. In this paper, the method of obtaining the optimal transformation order is the step-by-step search method. There are two traditional search algorithms: one is a two-dimensional search algorithm, and the other is a quasi-Newton search algorithm. Although the quasi-Newton search algorithm is less computationally expensive than the two-dimensional search algorithm, these two algorithms still cannot meet the actual real-time processing requirements of engineering. Therefore, we use the step-by-step search algorithm to replace the two-dimensional search algorithm, which greatly simplifies the calculation, and through a specific case, we explain the advantages of the step-by-step search method compared to other methods.

Case: The traditional two-dimensional search method uses the transformation order p as a variable to perform the scan search process; when the estimation accuracy of the parameter estimation is relatively high, in order to meet the accuracy requirements, a relatively small step size must be selected in the scan search process, and this will increase the computational complexity exponentially. For example, in the transformation order interval (0,2), if the search is performed with a step size of 0.0001, tens of thousands of FRFTs need to be performed, and the calculation amount is very large. Usually, we use the step-by-step search algorithm; that is, the first stage search is performed with a larger step size, and then the second stage search is performed with a smaller step size to find the maximum point, and so on until the estimation accuracy of the node objective function is satisfied. For example, we first searched for the maximum value in the transformation order interval (0,2) with a step size of 0.01, and then searched for the maximum value with a step size of 0.0001 in the interval corresponding to the maximum value after finding the maximum value. The amount was nearly halved. The step-by-step search method requires less computation and shorter computation time, so it has advantages over other methods. It can be concluded from the summary of the case that the step search method can effectively simplify the calculation amount and shorten the calculation time compared with other calculation methods, thereby improving the real-time performance of fault detection.

The existing methods for fractional-order fault detection often weaken the ability of traditional Fourier transform to diagnose faults. For this reason, this paper proposes an improved step-by-step search method, which makes the processing include both fractional-order faults and maintain better results in integer-order faults. The algorithm constructs an objective function $P(p,A,f_{0},f_{m})$; by solving the maximum point of the objective function, the order corresponding to the maximum point is the optimal transformation order of the FRFT. The objective function is essentially the peak value at the maximum energy concentration in the direction of maximum projection after the signal undergoes FRFT transformation. The algorithm consists of two stages: the first stage is to search on the transformation order interval (0,2) with a larger step size $\alpha_{l}$ and obtain the maximum value $P_{\max1}$ under the step size of $\alpha_{l}$, and the change order corresponding to the maximum value is $p_{1}$; after finding the maximum value, it enters the second stage and then calculates the maximum value of the objective function on the transformation order interval $\left[p_{1}-\alpha_{l},p_{1}+\alpha_{l}\right]$ with a smaller step size $\alpha_{s}$, and the change order corresponding to the maximum value is $p_{2}$, and so on, and the interval is gradually optimized. When the transformation order meets the estimation accuracy requirements, the transformation order at this time is the optimal transformation order. The steps of the algorithm are given below:

Phase 1:

(1) Let the initial interval of the transformation order p be (0,2);
(2) Construct the objective function $P(p,A,f_{0},f_{m})$, and its expression is as follows:
  $$P(p,A,f_{0},f_{m})=A\sqrt{\frac{1-j\cot\frac{p\pi }{2}}{2\pi }}\int_{-\infty }^{+\infty }exp(j\frac{\left(u^{2}+t^{2}\right)}{2}\cot\frac{p\pi }{2}-j\frac{ut}{\sin\frac{p\pi }{2}}+j2\pi f_{0}t+j\pi f_{m}t^{2})dt$$
(3) Input signal amplitude A, starting frequency $f_{0}$, and frequency modulation frequency $f_{m}$ and set the estimation accuracy $\alpha_{f}$;
(4) For *p* = 0 to 2, and choose a larger step size $\alpha_{l}$ to traverse the interval (0,2);
(5) Calculate the value of the objective function $P(p,A,f_{0},f_{m})$ and find the maximum value $P_{\max1}$ and its corresponding transformation order $p_{1}$;
(6) Calculate the value $P_{F}$ of the objective function $P(p,A,f_{0},f_{m})$ when the transform order of the signal *p* = 1;
(7) If the step size $\alpha_{l}$ at this time meets the requirements of the estimation accuracy $\alpha_{f}$, compare the values of $P_{\max1}$ with $P_{F}$. If $P_{F}$ is greater than $P_{\max1}$, then *p* = 1 is the optimal transformation order; if $P_{\max1}$ is greater than $P_{F}$, the transformation order in step (5) is the optimal transformation order; otherwise, it goes to phase 2.

Phase 2:

(1) Let the transformation interval of transformation order *p* be $\left[p_{1}-\alpha_{l},p_{1}+\alpha_{l}\right]$;
(2) Then take the smaller step size $\alpha_{s}$ and traverse on the transformation interval of step (1);
(3) Calculate the value of the objective function $P(p,A,f_{0},f_{m})$ and find the maximum value $P_{\max2}$ of the objective function in the interval and its corresponding transformation order $p_{2}$;
(4) If the step size $\alpha_{s}$ at this time meets the requirements of the estimation accuracy $\alpha_{f}$, compare the values of $P_{\max2}$ with $P_{F}$. If $P_{\max2}$ is greater than $P_{F}$, then the transformation order in step (3) is the optimal transformation order; if $P_{F}$ is greater than $P_{\max2}$, then *p* = 1 is the optimal transformation order. Otherwise, it is transferred to the next phase according to the analogy of the previous two phases until the step size in the phase satisfies the requirement of estimation accuracy $\alpha_{f}$, and the optimal transformation order of the FRFT is obtained.

## 4. Fault Diagnosis Method Based on SVM

At present, machine learning models, such as using logistic regression and SVM algorithms, are widely used to classify sample data [49]. Support vector machine, as a representative of shallow machine learning models, shows better generalization ability when classifying small sample data, and its model is relatively simple compared with other machine learning models, which is more convenient and effective for the research and model establishment of this article.

SVM is gradually developed based on the theory of statistical learning methods. It is a classifier that performs binary classification of sample data in a supervised learning manner. The goal of learning is to find a separating hyperplane in the feature space, which can classify samples into different classes and minimize the error of all samples from the hyperplane. When the sample to be classified is nonlinear, SVM uses a nonlinear mapping from the input space to the feature space to map the input to a feature vector, solving the problem of nonlinear sample space. The key point of the SVM is the determination of the optimal separation hyperplane and how to solve the nonlinear problem of the original sample space. The two key points will be introduced in detail below.

### 4.1. Determination of the Optimal Classification Hyperplane

**Figure 5:**
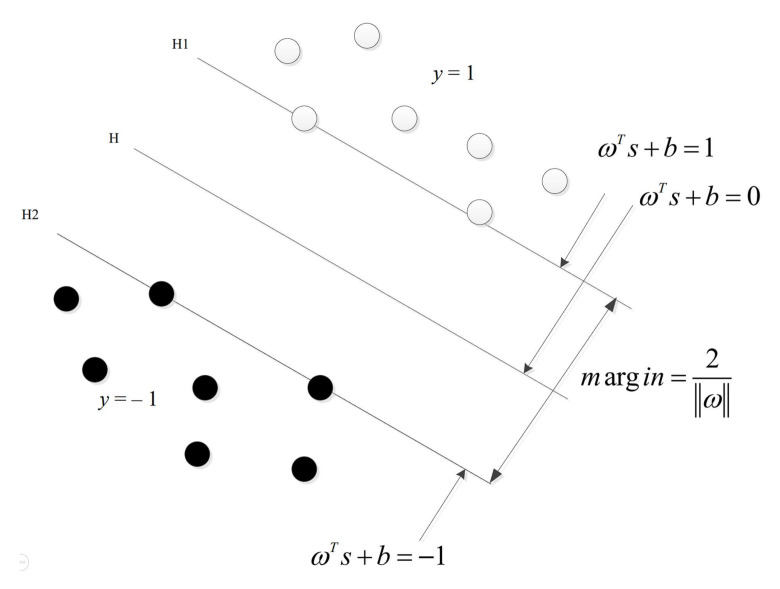
Schematic diagram of the maximum separation hyperplane.

Suppose given a training data set T on a feature space:

$T=\left\{\left(x_{1},y_{1}\right),\left(x_{2},y_{2}\right)\cdots \left(x_{t},y_{t}\right),\left(x_{N},y_{N}\right)\right\},t=1,2,3\ldots ,N$, where the sample $x_{t}\in R^{n}$ is the *t*-th feature vector, n is the sample data dimension, and $y_{t}\in \left\{-1,+1\right\}$ is the category. When $y_{t}=+1$, call $x_{t}$ a positive example; when $y_{t}=-1$, call $x_{t}$ a negative example, and $\left(x_{t},y_{t}\right)$ are called sample points. Generally, when the training data set is linearly separable, there are countless multiple separating hyperplanes that can correctly separate the two types of data, and the SVM uses the interval maximization strategy to find the optimal separation hyperplane (the maximum separation hyperplane); at this time, the solution is unique. The explanation of maximum interval is to find the hyperplane with the largest geometric interval for the training data set, which means to classify the training data with sufficient confidence and includes separating not only the positive and negative instance points, but also the closest point to the hyperplane. There is also enough certainty to separate them. Such a hyperplane has a good classification and prediction ability for unknown new instances. As shown in Figure 5, the white hollow points in the figure are the first type of instance points, representing the positive example points of $y_{t}=+1$; the black solid points in the figure are the other type of instance points, representing the negative example points of $y_{t}=-1$. H is a separating hyperplane, H1 and H2 are parallel and no instance point falls between them, and the separating hyperplane H is parallel to them and located in their center. These two parallel lines respectively pass through the sample points of the two types of samples in the training data set that are closest to the separation hyperplane. Such sample points are called support vectors. The distance between H1 and H2 is called the interval, and H1 and H2 are called the interval boundary. The mathematical expression of the separating hyperplane H in the figure is:

(13) $$w^{T}x+b=0$$

For the above linearly separable training data set T, construct and solve the constrained optimization problem:

(14) $$\underset{w,b}{\min}\frac{1}{2}||w|{|}^{2}$$

The constraints are:

(15) $$s.t.y_{t}\left(w^{T}x_{t}+b\right)\geq 1,t=1,2,3\ldots ,N$$

Find the optimal solution w and b, where w is the weight vector and b is the bias vector. The distance interval between H1 and H2 is $\frac{1}{2}||w|{|}^{2}$, so the maximum distance interval is equivalent to the minimum value of $||w|{|}^{2}$. In order to solve Equation (14), a Lagrangian function needs to be introduced. Through the corresponding variable conversion, the formula is as follows:

(16) $$L(w,b,a)=\frac{1}{2}||w|{|}^{2}-\sum_{t=1}^{N}a_{t}(y_{t}(w^{T}x_{t}+b)-1)$$

In the above formula, $a_{t}$ is the Lagrangian coefficient, $a_{t}\geq 0,t=1,2,3\ldots ,N$, the minimum value of Equation (16) is equivalent to the partial derivative w and b, and the partial derivative is zero. The final classification decision function considering the above constraints is:

(17) $$f(x)=sgn(\sum_{t=1}^{N}y_{t}a_{t}(x\cdot x_{t})+b_{0})$$

### 4.2. Nonlinear Problems in the Original Sample Space

The situation discussed above is under the premise that the original sample space can be linearly separable; when faced with the linear inseparability of the sample space, the data in the original space are mapped to the new space by selecting the appropriate nonlinear mapping. In the new space, the linear classification learning method is used to learn the classification model from the training data. This nonlinear mapping is called the kernel function, which is defined as follows:

(18) $$K(x_{i},x_{t})=\phi (x_{i})\cdot \phi (x_{t})(i,t=1,2,3\cdots ,N)$$

The kernel function selected by the SVM algorithm in this paper is the Gaussian radial basis function, and its mathematical expression is:

(19) $$K(x_{i},x_{t})=exp(-|x_{i}-x_{t}{|}^{2}/\sigma^{2})$$

Then, the final nonlinear classification decision function is:

(20) $$f(x)=sgn(\sum_{t=1}^{N}a_{t}{}^{*}K(x,x_{t})+b^{*})$$

In the face of linearly inseparable sample space, the original sample space data are nonlinearly mapped to the high-dimensional feature space through the kernel function, and at the same time, the problem of very large calculations in the high-dimensional space is solved, successfully achieving the learning of the classification model from the training data when the original sample space is linearly inseparable.

## 5. Experiments

### 5.1. Digital Simulation Test with Three Typical Signals

In order to verify that the FRFT has a strong ability to identify fractional-order faults in noise and complex background signals, several different simulation signals were listed in the experimental simulation. The following three basic signals were introduced:

The first signal is the classic chirp signal:

$x_{1}(t)=Aexp(j2\pi (f_{0}t+\frac{1}{2}f_{m}t^{2}))=exp(j2\pi (100t+\frac{1}{2}*5t^{2}))$, where the signal amplitude A=1, starting frequency $f_{0}=100$ Hz, frequency modulation frequency $f_{m}=5$ Hz, sampling frequency $f_{s}=1000$ Hz, and number of sampling points N=10,000.

The second signal is a sinusoidal signal $x_{2}\left(t\right)=sin\left(2\pi *100t\right)$, where the angular frequency w=100 Hz, sampling frequency $f_{s}=1000$ Hz, and number of sampling points N=10,000.

The third signal is a zero-mean Gaussian noise signal $n\left(t\right)$. When FRFT of a specific order is performed on the above signal, the peak energy concentration can appear in the fractional Fourier domain; while the Gaussian noise is in the fraction of any order, there will be no energy accumulation in the fractional Fourier domain. The above-mentioned characteristics of Gaussian noise can be used to simulate signal detection and parameter estimation under the noise background in the actual industry.

Different linear combinations of the above three different signals were performed to obtain three combined multicomponent signals:

(21) $$s_{1}(t)=exp(j2\pi (100t+\frac{1}{2}*5t^{2}))+sin\left(2\pi *100t\right)+n\left(t\right)$$

(22) $$s_{2}(t)=sin\left(2\pi *100t\right)+n\left(t\right)$$

(23) $$s_{3}(t)=exp(j2\pi (100t+\frac{1}{2}*5t^{2}))+n\left(t\right)$$

**Figure 6:**
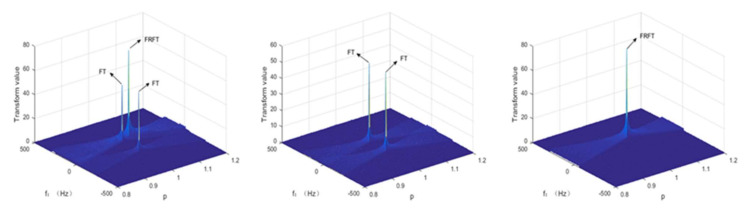
From left to right, FRFT processing of signals $s_{1}(t)$, $s_{2}(t)$, and $s_{3}(t)$.

Signal $s_{1}(t)$ simulates industrial fault signals that contain fractional signals, integer-order faults, and noise; signal $s_{2}(t)$ simulates fault signals that only contain integer-order faults and noise; and signal $s_{3}(t)$ simulates industrial fault signals that only contain fractional signals and noise. Next, FRFT was performed on the three multicomponent signals in sequence. The sampling frequency of the simulation signal used in the simulation experiment was $f_{s}=1000$ Hz, and the number of sampling points was N=10,000. Figure 6 shows the vibration analysis diagram of signals $s_{1}(t)$, $s_{2}(t)$, and $s_{3}(t)$ (from left to right). The peak value of the signal after FRFT and FT is marked in the figure. After the signal $s_{1}(t)$ is converted, there are both FT and FRFT values; after the signal $s_{2}(t)$ is converted, only the FT value exists, because the signal itself does not contain a fractional signal; after the signal $s_{3}(t)$ is converted, only the FRFT value exists, because the signal itself only contains the fractional signal. Analysis of the experimental results shows that the FRFT can accurately detect the chirp signal in a variety of mixed signals, which provides an effective feature extraction method for our subsequent experiments.

Then, for signal $s_{1}(t)$, the amplitude A of the chirp signal, the starting frequency $f_{0}$, and frequency modulation $f_{m}$ were changed, and the influence of each parameter change on the FRFT was analyzed. The specific results are shown in Table 1; p is the optimal order after FRFT, $f_{p}$ is the frequency at which energy is concentrated after FRFT, and $X_{p}(u)$ is the value after FRFT.

**Figure 7:**
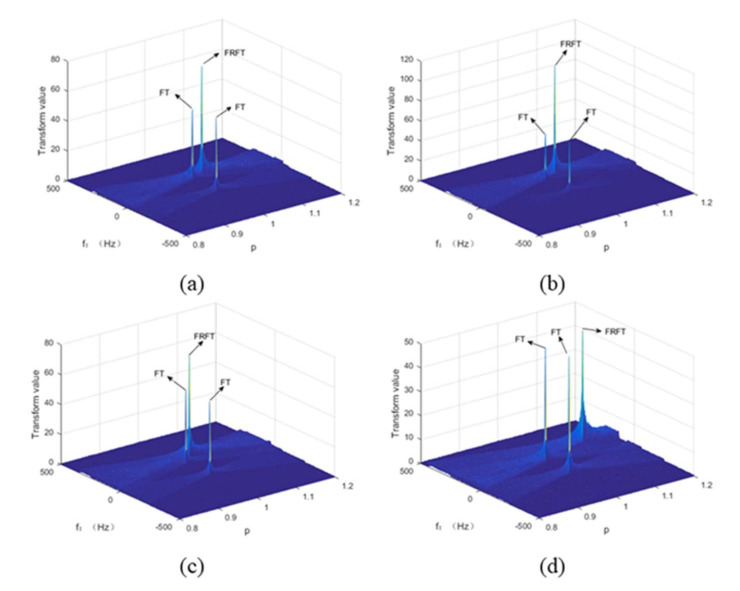
(**a**) Original signal; (**b**) changing A; (**c**) changing $f_{0}$; (**d**) changing $f_{m}$.

It can be seen from Figure 7 that the three parameters of amplitude A, starting frequency $f_{0}$, and FM frequency $f_{m}$ are correspondingly changed for an LFM signal, which has different effects on its FRFT. The change of A only affects the amplitude value at the energy accumulation; changing the $f_{0}$ parameter has an effect on the position of the energy concentration point of the FRFT, making the frequency and amplitude values of the energy concentration change; changing the $f_{m}$ parameter has an impact on the optimal order of the FRFT and the location of the energy concentration.

### 5.2. Actual Operating Data Test

**Figure 8:**
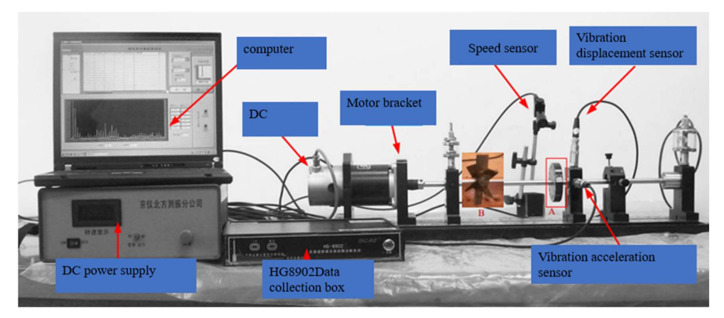
ZHS-2 type multifunctional motor test bench.

In order to verify the effectiveness of the algorithm proposed in this paper, we used the ZHS-2 multifunctional motor test bench with flexible rotors to conduct comparative simulation experiments. The motor test bench is shown in Figure 8. The parameters of the DC motor in the motor test bench are as follows: the rated power was 185 W, the rated voltage was 220 V, and the speed was 1500 r/min. A total of eight sensors were installed in the vertical and horizontal directions of the base to collect the vibration signals of the rotor. These vibration signals were transmitted by the HG-8902 data acquisition box, the equipment comes from Tang Xia Seiko Instrument Factory in Dongguan City, China. Six types of faults were considered in the experiment: rotor unbalance I (RU1), rotor unbalance III (RU3), rotor unbalance V (RU5), rotor unbalance VII (RU7), broken blades (PPB), and base loose (PL). In the specific diagnosis, the normal state and these six types of faults were distinguished. The first four types of faults were simulated by installing different numbers of screws on the rotor as shown in A in Figure 8, and the broken blades failure of the fan was realized by installing a fan with broken blades on the roller as shown in B in Figure 8.

The rotation speed of the motor was 1500 r/min, the sampling frequency was 1280 Hz, the acquisition time of each sample was 8 s, and 10,240 data points were recorded. In this experiment, 400 consecutive vibration acceleration signals were defined as one sample, 300 samples were collected for each type of fault, and a total of 1800 samples were collected to form a motor rotor data set. There were 300 samples of each type of failure data in the experimental data, and then we used the cross-validation method to divide the samples into the training set and the test set at a ratio of 2:1, and we selected 1200 sets of data as training samples for the construction of the classifier. The remaining 600 sets of data were used as test samples to test the performance of the diagnostic device constructed by the FRFT algorithm proposed in this paper.

During the experiment, we first performed the FRFT on the 1200 sets of samples of the training data set, and we input the extracted fractional fault feature values into the SVM model for training. The features of the fault diagnosis were the peak value of the maximum energy gathering point under the maximum projection direction of the signal in the fractional domain, that is, the small fault feature extracted by the FRFT of the signal in the fractional domain. After the training was completed, 600 sets of test samples were used to evaluate the accuracy of the constructed diagnostics model. The parameters of the SVM model were as follows: the penalty coefficient was 1, the kernel function type was RBF, the kernel function coefficient was auto, and the kernel function constant value was 0. At the same time, a comparison with the PCA processing of the fault data samples and input into the SVM diagnosis model and the classical Fourier transform processing of the fault data samples and input into the SVM model for training verified the effectiveness of this method for fractional-order faults.

**Figure 9:**
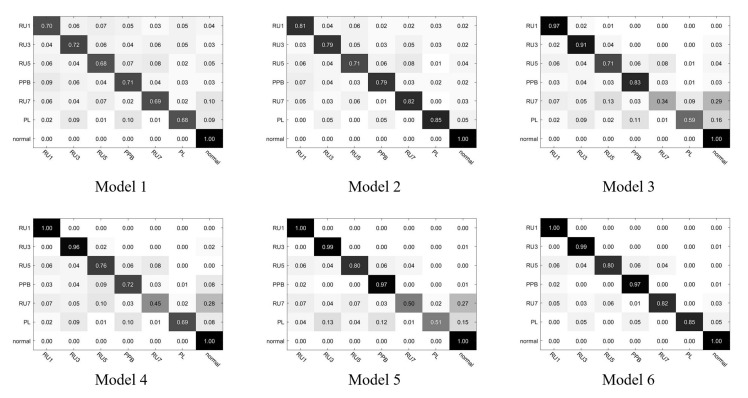
Confusion matrix information for the six tested models.

A total of six experiments were conducted in the whole simulation experiment. The first experiment was to perform PCA processing on the fault data samples and then input them into SVM for training. Experiment 2 was to perform FT processing on the fault data samples and then input them into SVM for training. Experiment 3 was to perform FRFT processing of transformation order *p* = 0.977 on fault data samples and input them into SVM for training. Experiment 4 was to perform FRFT processing of transformation order *p* = 1.133 on fault data samples and input them into SVM for training. Experiment 5 was to perform FRFT processing of the optimal transformation order on fault data samples and input them into SVM for training. Experiment 6 was a combined diagnosis method that used FRFT processing for fractional-order faults and FT processing for integer-order faults when processing signals that contain both fractional-order faults and integer-order faults and then input them into SVM for processing. The six experiments finally trained six models, namely model 1, model 2, model 3, model 4, model 5, and model 6, respectively, and model 6 is the optimization model finally proposed by the algorithm in this paper. Figure 9 shows the confusion matrix information obtained when the above six models were used for fault diagnosis.

In the experiment, six fault types and a type of normal data were used as the input of the model. These data were collected in a normal environment containing noise. The six fault types were RU1, RU3, RU5, RU7, PPB, and PL. Normal data were normal. From the confusion matrix in the figure, it can be seen that when classifying fault type data, in addition to accurately classifying the data, the model may misclassify the fault data as other faults or fail to detect faults. However, when the model diagnoses normal data, it will not misjudge the data as faulty data.

In addition, there are two important parameters for evaluating the model in the confusion matrix, namely recall and precision indicators. Their calculation formulas are as follows:

(24) $$precision=\frac{TP}{TP+FP}$$

(25) $$recall=\frac{TP}{TP+FN}$$

In the above formula, TP represents correctly predicting the instance as a positive (true instance), FP represents mispredicting an instance as positive (false positive), and FN represents incorrectly predicting an instance as a counterexample (false counterexample). Through the specific confusion matrix information in Figure 9, we can obtain the specific values of these two parameters of these confusion matrices, and the results are shown in Table 2.

From the content of Table 2, we can better analyze the quality of the confusion matrix through these two parameter indicators, so as to judge the quality of the trained model. The recall and precision indicators of each model shown in the table are the average of the recall and precision indicators of each class of the model. As a final result, it can be concluded that both recall and precision indicators of Model 6 are the highest, and model 6 is the optimization model finally proposed by the algorithm in this paper.

Table 3 shows the accuracy rates of model 2 to model 5 on the test sets of various fault data. Comparing the results of model 2 and model 5, it can be found that the diagnostic accuracy for the first four types of faults in the model 5 pair table is higher than that of model 2. The improvement on the data types RU7 and PL is not good. The reason is that when dealing with both fractional-order and integer-order faults, model 5 only has a better effect on fractional-order faults, while model 2 has a better effect on integer-order failures but is worse than model 5 on fractional-order failures. Comparing the results of model 2, model 3, and model 4, this paper added two sets of control experiments that used fixed transformation order to process fault signals, and it is concluded that the fractional fault diagnosis model under the nonoptimal transformation order is also better than the fault diagnosis model using the classic Fourier transform, which makes the method applicable to industry with better real-time performance.

For this reason, model 6 proposed by the improved algorithm of this paper was added to the experiment and compared with model 1, model 2, and model 5; the results are shown in Table 4. Model 6 has relatively good effects on six different types of faults, indicating that the improved method solves the problem of fractional-order fault detection that often weakens the ability of traditional Fourier transform to diagnose faults.

### 5.3. Conclusions

Table 5 shows the classification methods and classification accuracy of all models in the experiment. The four fault types RU1, RU3, RU5, and RU7 in Table 5 contain integer-order faults, while the two fault types PPB and PL contain fractional-order faults. The following are the definitions and descriptions of models 1 to 6.

Model 1: The classification method is PCA + SVM. The model first performs PCA processing on the input fault data to obtain the converted value and then inputs the obtained value into the SVM for training to obtain the final model.

Model 2: The classification method is FT + SVM. The model first performs FT processing on the input fault data to obtain the converted value and then inputs the obtained value into the SVM for training to obtain the final model.

Model 3: The classification method is FRFT (*p* = 0.977) + SVM. The model first performs FRFT processing with transformation order *p* = 0.977 on the input fault data to obtain the converted value and then inputs the obtained value into the SVM for training to obtain the final model.

Model 4: The classification method is FRFT (*p* = 1.133) + SVM. The model first performs FRFT processing with transformation order *p* = 1.133 on the input fault data to obtain the converted value and then inputs the obtained value into the SVM for training to obtain the final model.

Model 5: The classification method is FRFT (*p* is optimal transformation order) + SVM. The model first performs FRFT processing with optimal transform order on the input fault data to obtain the converted value and then inputs the obtained value into the SVM for training to obtain the final model.

Model 6: The classification method is FRFT + FT + SVM. The model performs FT processing on the fault data including integer-order faults, performs FRFT processing on fractional-order faults with the optimal transformation order, and then inputs the value obtained after processing into the SVM model to obtain the final model.

The classification method used in model 1 is PCA + SVM. It can be seen from the table that the diagnostic accuracy of this method for integer-order faults and fractional-order faults is relatively general, indicating that the PCA method has a general diagnostic effect on integer-order and fractional-order faults. The classification method used in model 2 is FT + SVM. It can be seen from the table that this method has improved the diagnostic accuracy for integer-order faults and fractional-order faults compared with model 1, and it can have a good effect on diagnosing whether the fault is fractional-order or integer-order. The classification method used in model 3 is FRFT (*p* = 0.977) + SVM, and the transformation order of this method is fixed at 0.977 when FRFT is performed on the fault data. It can be seen from the table that the diagnostic accuracy of this method for fractional-order faults is higher than that of model 2, but the accuracy for integer-order faults is worse than that of model 2. The classification method used in model 4 is FRFT (*p* = 1.133) + SVM, and the transformation order of this method is fixed at 1.133 when FRFT is performed on the fault data. It can be seen from the table that the diagnostic accuracy of this method for fractional-order faults is higher than that of model 2, but the accuracy for integer-order faults is worse than that of model 2. The FRFT transformation orders adopted by model 3 and model 4 are not optimal transformation orders. The reason for comparing model 2, model 3, and model 4 is to verify that the FRFT method under the nonoptimal transformation order can also have a better diagnosis effect than the PCA and FT methods in diagnosing fractional-order faults, so as to verify that the FRFT method has a certain real-time fault diagnosis. The classification method used in model 5 is FRFT+SVM, and the transformation order of this method is the optimal transformation order when FRFT is performed on the fault data. It can be seen from the table that the diagnostic accuracy of this method for fractional-order faults is higher than that of model 2, but the accuracy for integer-order faults is poorer than that of model 2, indicating that the traditional FRFT method has certain defects; it has a better detection effect on fractional-order faults, but the detection effect on integer-order faults is not as good as that of the FT method. The classification method used in model 6 is the new method proposed in this paper, namely FRFT + FT + SVM. When diagnosing fault signals containing both integer-order faults and fractional-order faults, the method combines the two methods, using the FRFT method to handle fractional-order faults and the FT method to handle integer-order faults to obtain a model that has good detection performance for all fault types. Comparing model 2, model 5, and model 6 in the table, it can be found that model 6 has a greater improvement in handling fractional-order faults and integer-order faults than model 2 and model 5, and the same is true for other models. Therefore, we can conclude that the classification method proposed in this paper makes up for the deficiency of the traditional FRFT method having a good detection effect on fractional-order faults but a poor detection effect on integer-order faults. In the face of fault signals containing both integer-order faults and fractional-order faults, the whole has a good diagnostic effect.

## 6. Summary and Prospect

Summary: The new method built in this paper has provided a new idea for the mixed fault of a motor in the time domain and frequency domain. Motor equipment is often interfered with by complex environmental noise during operation. The fault characteristic frequency is often a nonstationary signal, and the fractional-order small fault signals are easily hidden in strong noise, which makes the existing frequency domain diagnosis methods dominated by integer-order methods which are unable to detect fractional-order fault signals effectively. The new method introduces the fractional Fourier transform method based on the time domain and the frequency domain, which can transform the signal in the maximum projection direction in the fractional domain and obtain the peak energy concentration in the projection direction, which is the signal in the maximum projection direction. The tiny fault features in the fractional domain effectively separate the fault feature signal from the strong noise, which cannot be detected by other methods. The fault diagnosis method built in this paper firstly inputs the extracted fault features and the prior information of the corresponding fault category into the SVM classification model and then trains a more accurate and efficient fault classifier through the set cost function. It not only has a strong detection ability for fractional-order tiny faults but also has a good detection effect on traditional integer-order faults, so it has strong robustness to the classification ability of both integer-order and fractional-order frequency domain faults.

Prospect: While constructing the optimal projection direction model in this paper, more prior information is needed to determine the optimal projection direction. Therefore, determining how to reduce the dependence on prior information during determining the optimal direction and constructing a data-driven multilevel and multilayer fault detection classification method according to the size of the fault, in order to ensure the online detection and classification of motor faults, is a research direction to be focused on in the future. At the same time, industrial motors often show the phenomenon of multiple types of faults occurring at the same time; electrical equipment not only has frequency domain type faults, but also amplitude type and phase type faults. The concurrent occurrence of these multiple types of faults causes great difficulties in motor fault diagnosis. When the structure of the neural network is getting deeper and deeper, the gradient descent algorithm can be replaced by the Kalman filter, and the Kalman filter is used to adaptively update the neural network [50,51,52,53]. Therefore, this is also an important direction for future research in the field of faults.

## Figures and Tables

**Table 1:** The effect of chirp signal parameters on FRFT.

Chirp Signal Parameters			Energy Concentration Position after FRFT (Peak Point)		
A	$f_{0}/\mathrm{Hz}$	$f_{m}/\mathrm{Hz}$	p	$X_{p}(u)/\mathrm{Hz}$	$f_{p}/\mathrm{Hz}$
1	100	5	1.0320	75.1522	124.80
1.5	100	5	1.0320	112.8217	124.80
1	150	5	1.0320	69.8297	174.70
1	100	20	1.1260	48.1132	196.10

**Table 2:** Precision and recall information for six confusion matrices.

Test Model	Precision	Recall
Model 1	74%	69%
Model 2	79%	71.5%
Model 3	81%	72.5%
Model 4	82%	76.3%
Model 5	84%	79.5%
**Model 6**	**92%**	**90.5%**

**Table 3:** The accuracy of the test set in each model in the simulation experiment.

Fault Data Type	Model 2	Model 3	Model 4	Model 5
RU1	81%	97%	**100%**	**100%**
RU3	79%	91%	**96%**	**99%**
RU5	71%	71%	**76%**	**80%**
PPB	79%	83%	**72%**	**97%**
RU7	82%	34%	**45%**	**50%**
PL	85%	59%	**69%**	**51%**

**Table 4:** The accuracy of the test set in each model in the simulation experiment.

Fault Data Type	Model 1	Model 2	Model 5	Model 6
RU1	70%	81%	**100%**	**100%**
RU3	72%	79%	**99%**	**99%**
RU5	68%	71%	**80%**	**80%**
PPB	71%	79%	**97%**	**97%**
RU7	69%	82%	**50%**	**82%**
PL	68%	85%	**51%**	**85%**

**Table 5:** Classification accuracy of all experimental models.

Experimental Model	Classification Method	RU1	RU3	RU5	PPB	RU7	PL
Model 1	PCA + SVM	70%	72%	68%	71%	69%	68%
Model 2	FT + SVM	81%	79%	71%	79%	82%	85%
Model 3	FRFT (*p* = 0.977) + SVM	97%	91%	71%	83%	34%	59%
Model 4	FRFT (*p* = 1.133) + SVM	100%	96%	76%	72%	45%	69%
Model 5	FRFT (*p* is optimal transformation order) + SVM	100%	99%	80%	97%	50%	51%
**Model 6**	**FRFT + FT + SVM**	**100%**	**99%**	**80%**	**97%**	**82%**	**85%**

## Data Availability

Available online: https://engineering.case.edu/bearingdatacenter/welcome (accessed on 1 February 2022).
